# Evaluation of the Efficacy and Safety of Single‐Needle Radiofrequency in Patients With Facial Sebaceous Hyperplasia: A Retrospective Study

**DOI:** 10.1111/jocd.70472

**Published:** 2025-09-25

**Authors:** Xiaoyan Qiu, Yangyang Qiu, Meiqing Chen, Ling Huang, Xinyu Lin, Yi Wei, Ji Yang, Yuting Chen, Lujuan Gao

**Affiliations:** ^1^ Department of Dermatology Zhongshan Hospital (Xiamen), Fudan University Xiamen China; ^2^ Department of Dermatology Zhongshan Hospital, Fudan University Shanghai China

**Keywords:** dermoscopy, sebaceous hyperplasia, single‐needle radiofrequency

## Abstract

**Background and Aims:**

Sebaceous hyperplasia is a prevalent comorbidity in dermatology. However, there is a notable absence of an effective and safe treatment modality in current clinical practice. This study aimed to evaluate the efficacy and safety of single‐needle radiofrequency for treating facial sebaceous hyperplasia.

**Methods:**

A retrospective study was undertaken, enrolling patients diagnosed with facial sebaceous hyperplasia that spanned from December 2021 to August 2023 in the department of dermatology, Zhongshan Hospital (Xiamen), Fudan University. Dermoscopy was employed for the objective evaluation before treatment and at 4‐week intervals following each treatment session. The Pain Visual Analogue Scale was assessed by each patient immediately after treatment. The treatment endpoint was defined as the absence of more than 50% visible white‐yellowish globules under dermoscopy. Treatment responses were classified as optimal, good, and poor, determined by the regression rate of skin lesions, set at over 90%, 50%–90%, and below 50%, respectively. All patients were subsequently subjected to follow‐up examinations every 4 weeks for a period of 6 months, along with an assessment of any complications. Patients underwent systematic follow‐up assessments at 24–72 h for acute complications and every 4 weeks for 6 months to evaluate long‐term outcomes.

**Results:**

A total of 45 patients were included, with a male‐to‐female ratio of 1.8:1. The mean age of onset was 42.9 ± 9.6 years. Forty‐four patients (97.8%) achieved optimal outcomes, while one patient (2.2%) showed a good result. The average number of treatments required was 2.2 ± 0.8. Male patients required more treatment sessions and a wider pulse width compared to female patients (*p* < 0.05). During the follow‐up, only one case (2.2%) experienced a relapse. Complications following treatment included redness (100%) and crusting (8.9%). The average treatment pain score was recorded as 2.6 ± 0.5.

**Conclusion:**

The use of single‐needle radiofrequency proves to be an effective and safe treatment for patients with sebaceous hyperplasia. Dermoscopy emerges as a valuable auxiliary tool for evaluation.

## 1 Introduction

Sebaceous hyperplasia (SH) manifests as benign lesions predominantly affecting the face, occurring in approximately 1% of the healthy population. Typically, it manifests as skin‐colored or whitish‐yellow papules with a normal umbilical appearance, varying in size from 2 to 9 mm [1]. SH can have significant psychological impacts and raise cosmetic concerns for patients due to their primary occurrence in the facial area, affecting their self‐image [2].

Historically, treatment options for SH have encompassed cryosurgery, photodynamic therapy, laser therapy, and topical trichloroacetic acid [3]. A variety of lasers have been employed in the treatment of SH, each utilizing distinct mechanisms. Argon and CO_2_ lasers function ablatively by mechanically destroying SH lesions. The 585 nm pulsed dye laser (PDL) operates within the visible (yellow) spectrum, targeting oxyhemoglobin to achieve selective photothermolysis of dermal vasculature. Meanwhile, the Diode laser primarily targets water, generating heat at depths of 100 to 600 μm in the dermis, thereby inducing thermal destruction of sebaceous gland structure and function [2]. However, these approaches inevitably induce surface damage, leading to temporary side effects such as erythema, crusting, and discomfort. Additionally, there exists a potential risk of scarring, hyperpigmentation, dyspigmentation, and recurrence of the lesions [2, 4]. Oral isotretinoin has also been utilized for SH treatment. Nevertheless, previous studies have indicated a high relapse rate in patients not consistently maintained on therapy [5].

Kobayashi et al. [6] histologically confirmed that one needle insertion and high‐frequency current application possessed a selective electrothermal decomposition effect on sebaceous glands, capable of reducing their number. The damaged sebaceous glands were replaced by fibrous tissue, and this phenomenon remained observable even after 6 months of radiofrequency treatment. In this study, we explored the application of single‐needle radiofrequency for the treatment of facial SH, with a focus on evaluating the efficacy and safety of this novel approach.

## 2 Materials and Methods

### 2.1 Study Population

This retrospective study was conducted in the department of dermatology, Zhongshan Hospital (Xiamen), Fudan University, Xiamen, China, after obtaining approval from the hospital's ethics committee. The approval number is B2024‐002. As routine clinical practice, informed consent was obtained from all patients before invasive treatment. Patients diagnosed with facial SH by two experienced dermatologists, based on typical clinical manifestations (skin‐colored or whitish‐yellow papules with a normal umbilical appearance [1]) and dermoscopic features (pathognomonic white‐yellow globules with crown vessels [7]), and who underwent single‐needle radiofrequency treatment between December 2021 and March 2023, were enrolled. Histopathological examinations were performed when required. Patients with implanted electronic devices, metal objects, or skin‐filling materials were excluded from this study. Common head and facial comorbidities, including androgenic alopecia, seborrheic dermatitis, and rosacea, were documented before treatment.

### 2.2 Single‐Needle Radiofrequency Treatment

**FIGURE 1:**
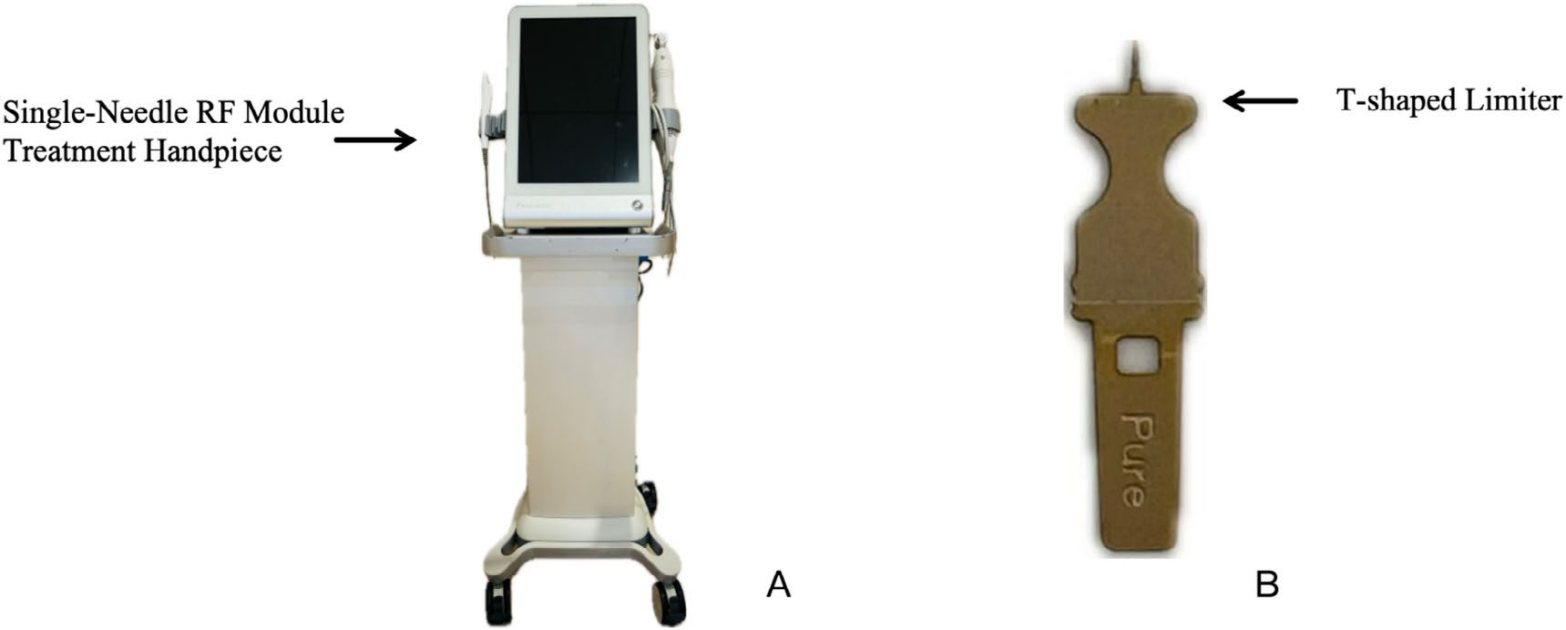
High‐frequency electrocautery device (Shenzhen Peninsula Medical Co. LTD) (A) and the single‐needle radiofrequency probe (B).

**FIGURE 2:**
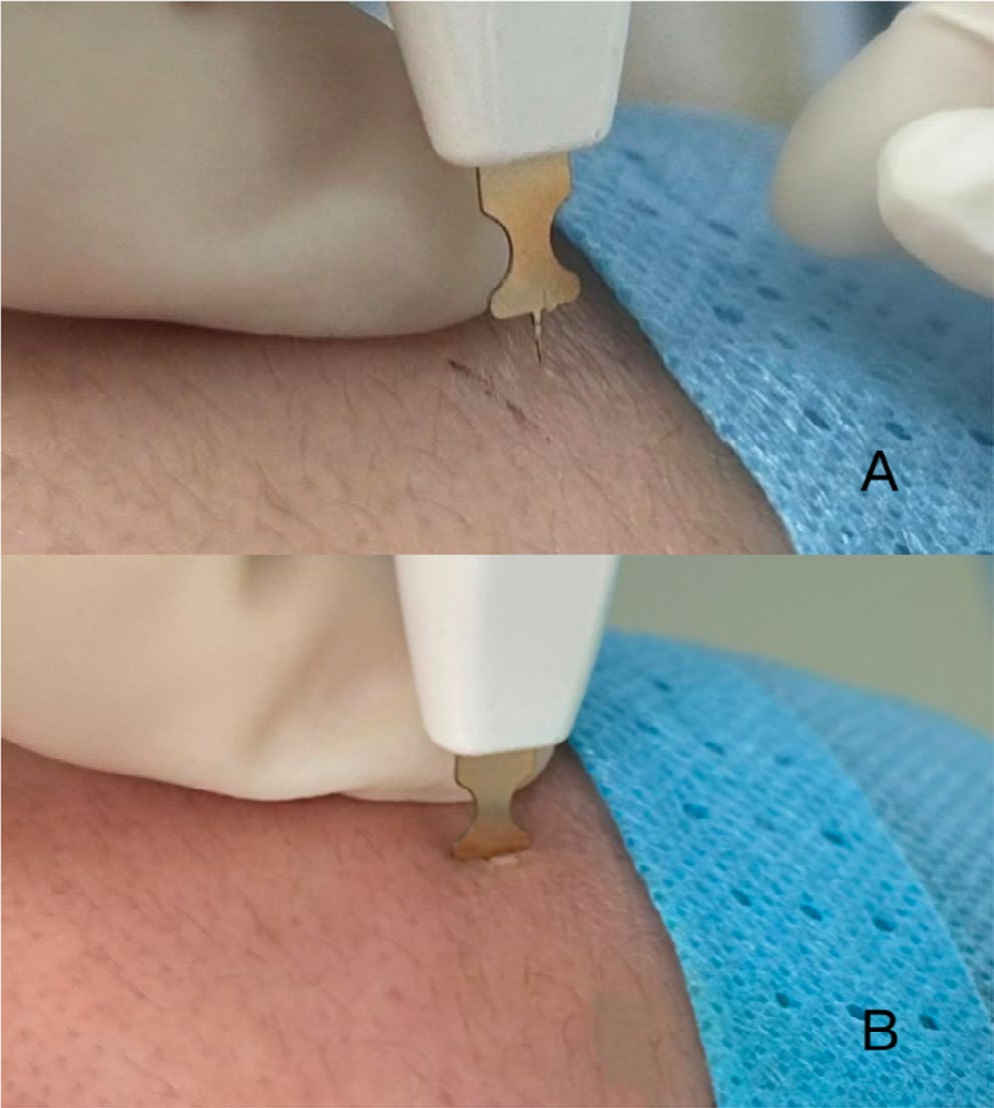
Single‐needle radiofrequency treatment procedure: Disinfect the skin lesion, then insert the needle vertically (A) and precisely deliver energy to the target sebaceous gland (B).

Single‐needle radiofrequency using a high‐frequency electrocautery device (Shenzhen Peninsula Medical Co. LTD., Model Unicorn I, 201820101997, Figure 1A) was employed for the treatment. The single‐needle radiofrequency probe utilized in this study features a T‐shaped design (Figure 1B). The T‐shaped limiter creates a barrier on the skin surface during insertion, stabilizing the insertion depth. The proximal end of the probe is insulated, enabling precise destruction of sebaceous glands while sparing the epidermis [8]. Depending on the size of the lesions, a needle with a length ranging from 1.5 to 2.5 mm was carefully chosen. The power settings were configured to 3–4 W, and the pulse width ranged from 300 to 350 ms. The energy delivery therapy was administered at the central catheter opening of the lesion (Figure 2), with an additional 4–6 treatments performed around the lesion (360°), spaced 1 mm apart. The patients underwent treatment at 4‐week intervals, with the total number of sessions individualized based on their treatment response.

### 2.3 Evaluation of Treatment Response

The objective assessments of treatment efficacy via dermoscopy were conducted by two experienced dermatologists who performed a global evaluation based on lesion number. Dermoscopic images were captured using the FotoFinder Medicam 800HD (FotoFinder, Germany) before treatment and at each follow‐up visit after treatment. The treatment endpoint was defined as the absence of more than 50% visible white‐yellowish globules under dermoscopy. Treatment responses were categorized as optimal, good, and poor, corresponding to regression rates of skin lesions ≥ 90%, 50%–90%, and < 50%, respectively. Patients were monitored at multiple timepoints. Pain was assessed via the Pain Visual Analogue Scale (VAS) immediately after each treatment. Acute complications such as redness, swelling, and incrustation were evaluated at 24–72 h post‐procedure. For the recurrence of rash and other complications including cicatrix, pigmentation, and hypopigmentation, patients were followed up every 4 weeks for 6 months following the last treatment.

### 2.4 Statistical Analysis

Descriptive statistics were presented as means and standard deviations for numerical variables, while categorical variables were expressed as both numerical values and percentages. Group comparisons for measures adhering to a normal distribution were conducted using two‐sided independent samples *t*‐tests or one‐way ANOVA. The statistical significance of continuous variables was assessed using the Spearman correlation coefficient. A significance level of *p* < 0.05 was considered statistically significant. Statistical analyses were performed using SPSS (version 25.0; SPSS Inc., Chicago, IL, USA).

## 3 Results

In this study, a total of 45 patients were included (Tables 1 and 2), exhibiting a male‐to‐female ratio of 1.8:1. The mean age of onset was 42.9 ± 9.6 years, ranging from 22 to 70 years. The average course of the disease was 3.8 ± 2.6 years, ranging from 0.5 to 12 years. For males, the mean course of the disease was 4.4 ± 2.8 years, while for females, it was 2.6 ± 1.6 years. The difference in the course of the disease between males and females was 1.8 years, with a 95% confidence interval (95% CI) ranging from 0.3 to 3.4 years (*p* < 0.05), indicating a statistically significant longer course in males. Regarding the average number of lesions, it was found to be 13.0 ± 10.5, ranging from 1 to 35. Male patients in the present study had an average of 15.8 ± 10.7 lesions, while females presented with 7.9 ± 8.3 lesions. The difference in the number of lesions between males and females was 7.9, with a 95% CI ranging from 1.7 to 14.1 (*p* < 0.05), indicating that males had significantly more lesions. Lesion distribution was as follows: forehead (55.6%), temporalis (24.4%), cheek (84.4%), and chin (4.4%). Fitzpatrick subtype III was identified in 40 cases (88.9%), whereas five cases (11.1%) were classified as Fitzpatrick subtype IV. Comorbidities were observed in some cases, with androgenic alopecia affecting 60%, seborrheic dermatitis in 4.4%, and rosacea in 2.2% of the patients. It's noteworthy that androgenic alopecia was prevalent among males, affecting 27 cases (93.1%).

**TABLE 1:** Treatment of 45 patients with sebaceous hyperplasia.

Features	Value
Gender	
Male	29 (64%)
Female	16 (36%)
Onset age (year)	42.9 ± 9.6 (22–70)
Course (year)	3.8 ± 2.6 (0.5–12)
Number of lesions	13.0 ± 10.5 (1–35)
Distribution of lesions	
Forehead	25 (55.6%)
Temporalis	11 (24.4%)
Cheek	38 (84.4%)
Chin	2 (4.4%)
Fitzpatrick subtype	
III	40 (88.9%)
IV	5 (11.1%)
Comorbidity	
Androgenic alopecia	27 (60%)
Seborrheic dermatitis	2 (4.4%)
Rosacea	1 (2.2%)
Therapeutic parameter	
Power (w)	3.1 ± 0.3 (3–4)
Pulse width (ms)	317.2 ± 32.3 (250–350)
Number of treatments	
Average number of treatments	2.2 ± 0.8 (1–3)
1	9 (20.0%)
2	17 (37.8%)
3	19 (42.2%)
Pain score (VAS)	2.6 ± 0.5 (2–4)
Complications	
Red and swollen	45 (100%)
Incrustation	4 (8.9%)
Cicatrix	0 (0%)
Pigmentation	0 (0%)
Hypopigmentation	0 (0%)
Treatment effect	
Optimal	44 (97.8%)
Good	1 (2.2%)
Poor	0 (0%)
Relapse in 6 months	
Yes	1 (2.2%)
No	44 (97.8%)

**TABLE 2:** Gender difference in clinical treatment of 45 patients with sebaceous hyperplasia.

Features	Male	Female	Mean difference (95% CI)	*p*
Onset age (year)	43.2 ± 8.6	42.4 ± 11.4	0.8 (−5.2, 6.9)	0.78
Course (year)	4.4 ± 2.8	2.6 ± 1.6	1.8 (0.3, 3.4)	0.02
Number of lesions	15.8 ± 10.7	7.9 ± 8.3	7.9 (1.7, 14.1)	0.01
Number of treatments	2.5 ± 0.7	1.8 ± 0.8	0.6 (0.2, 1.1)	0.006
Therapeutic parameter				
Power (w)	3.1 ± 0.3	3.1 ± 0.3	0.02 (−0.1, 0.2)	0.774
Pulse width (ms)	325.9 ± 29.5	301.6 ± 32.2	24.3 (4.4, 44.2)	0.014

**FIGURE 3:**
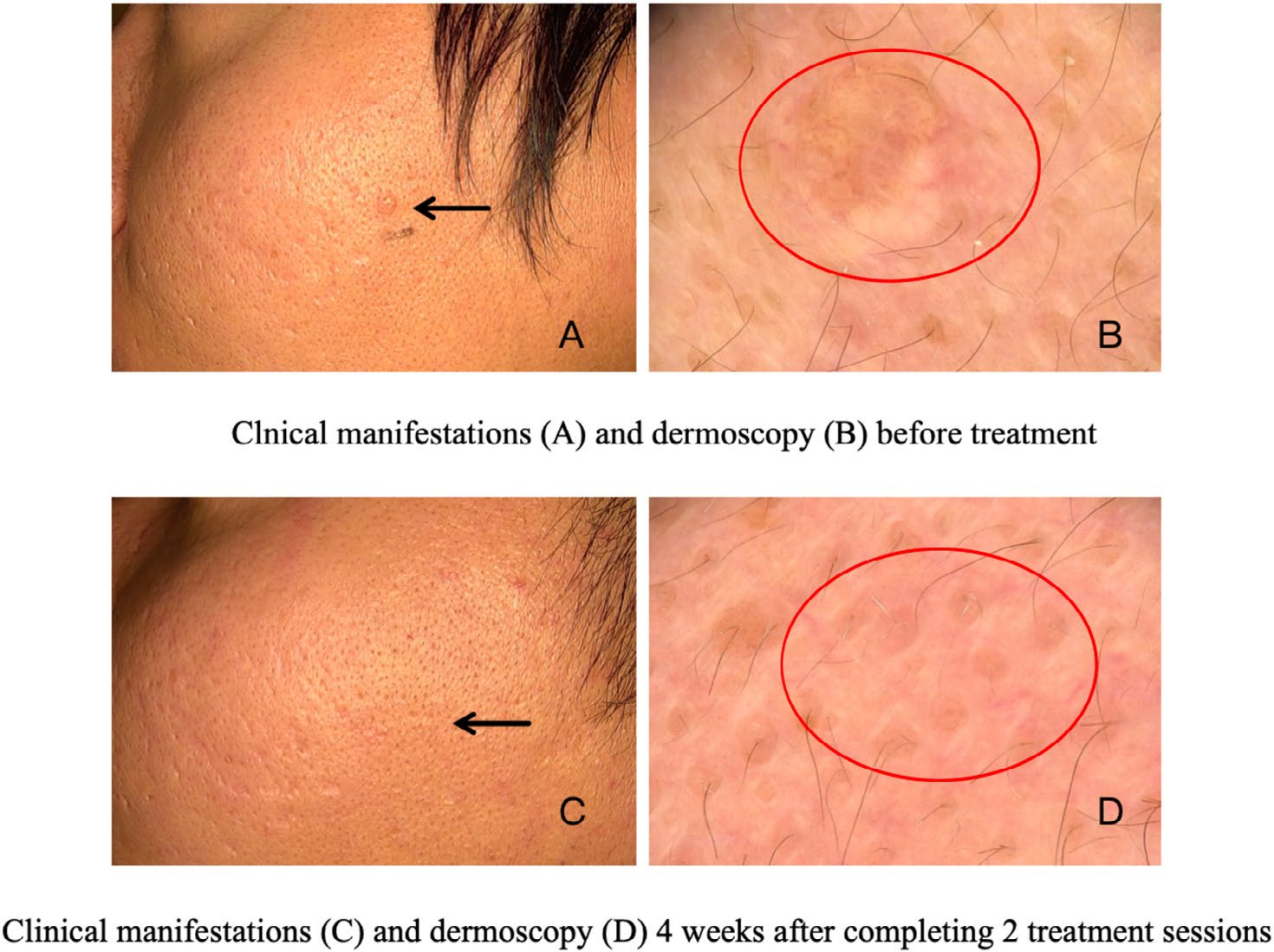
Sebaceous hyperplasia: pre‐ and post‐single‐needle radiofrequency. (A) Pretreatment: flesh‐colored umbilicated papules (black arrow). (B) Dermoscopy (20×): white‐yellowish globules and crown vessels (red circle). (C) Post‐treatment (4 weeks after completing 2 treatment sessions): Lesion sussided (black arrow). (D) Dermoscopy (20×): sebaceous structures resolved (red circle).

**FIGURE 4:**
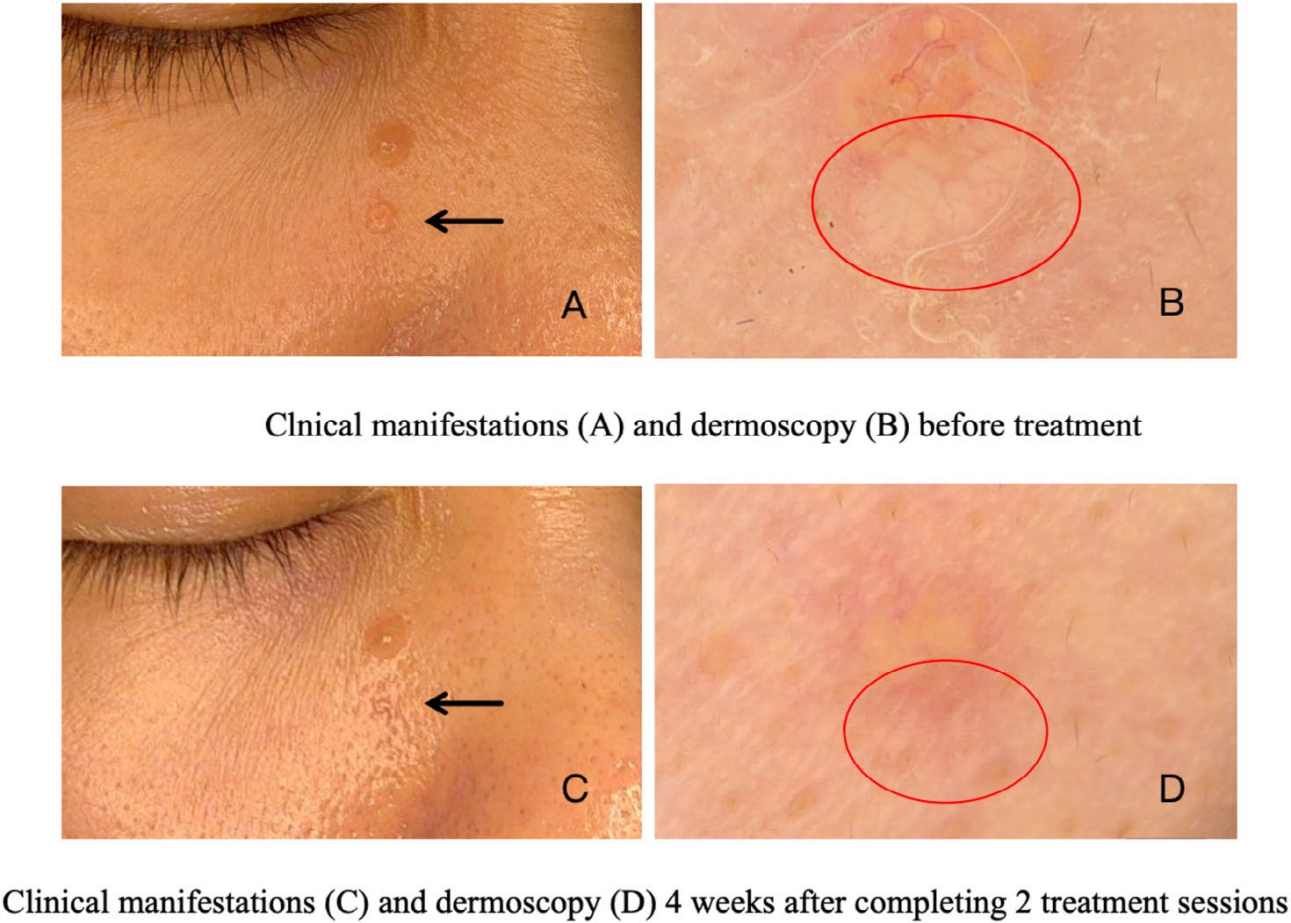
Sebaceous hyperplasia: pre‐ and post‐single‐needle radiofrequency. (A) Pre‐treatment: flesh‐colored umbilicated papules (black arrow). (B) Dermoscopy (20×): white‐yellowish globules and crown vessels (red circle). (C) Post‐treatment (4 weeks after completing 2 treatment sessions): lesion sussided (black arrow). (D) Dermoscopy (20×): sebaceous structures resolved (red circle).

**FIGURE 5:**
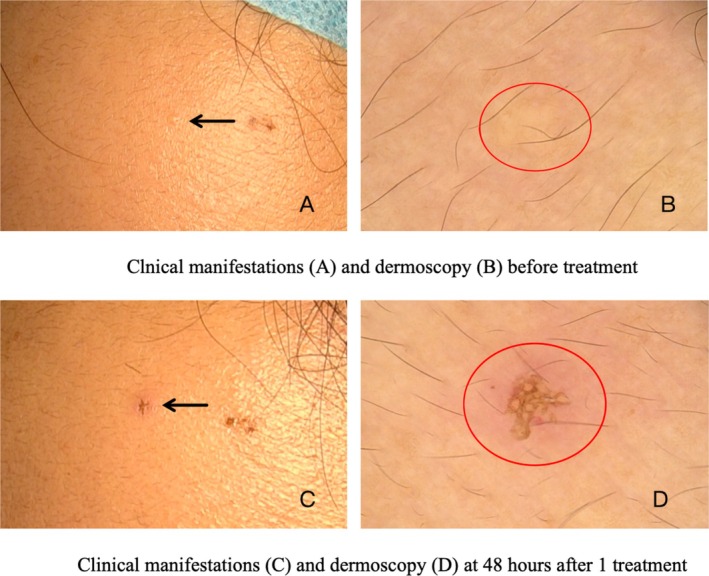
Inflammatory response phase after single‐needle radiofrequency treatment. (A) Pre‐treatment: yellow papules (black arrow). (B) Dermoscopy (20×): yellowish globules (red circle). (C) Post‐treatment (48 h after 1 treatment): redness and crusting of the lesion (black arrow). (D) Dermoscopy (20×): redness and crusting (red circle).

Evaluation of treatment response using dermoscopy (Figures 3 and 4) revealed that 44 patients (97.8%) achieved optimal outcomes, while one patient (2.2%) showed a good result. The average number of treatments required to achieve the endpoint was 2.2 ± 0.8 (1–3). Males underwent an average of 2.5 ± 0.7 treatments, whereas females had an average of 1.8 ± 0.8 treatments. The number of treatments for males was significantly higher than that for females (*p* < 0.05). The average power of the single‐needle radiofrequency treatment was 3.1 ± 0.3 W (3–4 W). The average pulse width was 317.2 ± 32.3 ms (250–350 ms). The pulse width for males was 325.9 ± 29.5 ms, while for females, it was 301.6 ± 32.2 ms. The difference in pulse width between males and females was 24.3 ms, with a 95% CI ranging from 4.4 to 44.2 ms (*p* < 0.05), indicating a statistically significant wider pulse width in males. Additionally, the number of treatments exhibited a positive correlation with the duration of the patient's disease (*p* < 0.05, *r* = 0.4) and the number of lesions (*p* < 0.001, *r* = 0.5). The average treatment pain score was recorded as 2.6 ± 0.5 (2–4). Recurrence occurred in only one patient (2.2%) 4 months posttreatment. Complications following treatment included redness (100%) and crusting (8.9%) (Figure 5), both of which resolved within 2–3 days without resulting in scarring, hyperpigmentation, or hypopigmentation.

## 4 Discussion

In SH, the sebaceous glands exhibit an increase in both size and number, yet maintain a normal structure [9]. Sebaceous glands, integral components of the skin, are subject to the influences of intrinsic and extrinsic aging factors, leading to distinct clinical features and histological changes. Extrinsic factors affecting sebaceous glands primarily involve sun exposure and the use of immunosuppressants. Sun exposure has the direct capability to activate signaling pathways, such as the Wnt/β‐catenin, Notch, AhR, and p53 pathways, potentially associated with sebaceous gland dedifferentiation and hyperplasia [3, 10]. Our study revealed that the majority of SH lesions manifest on the forehead and cheek areas, which were in accordance with the anatomical distribution location of sebaceous glands. In addition, the predilection of SH in the facial area instead of the chest or back, in some instances, supports the notion of sun exposure as a contributing factor to the development of this dermatological condition [3, 10].

Intrinsic age‐related hormonal changes, primarily involving androgens, play a significant role in the development of sebaceous gland [7]. Sebaceous cells express hormone receptors extensively, primarily regulating sebaceous gland activity by binding to dihydrotestosterone [8]. Serum testosterone levels decrease with age in both males and females [11]. Reduced androgen levels contribute to decreased cellular turnover in the aged sebaceous glands on the face, resulting in glandular hyperplasia [10]. The mean age of onset for patients in our study was 42.9 years old. The proportion of male patients was higher, along with an increased course and number of lesions. Additionally, all patients with androgenetic alopecia were male. A previous study has demonstrated that the sebaceous glands of patients with androgenetic alopecia exhibit specific binding proteins [12]. Furthermore, the sebaceous glands of patients with androgenetic alopecia displayed a higher affinity for dihydrotestosterone compared to those of the general population, potentially leading to the overgrowth of the sebaceous glands [12, 13].

SH is at risk of recurrence after treatment, and the demand to remove the entire sebaceous glands is challenging, highlighting the necessity for a safer and more effective treatment method. Intralesional electrodesiccation has emerged as an alternative, with Bader et al. [14] reporting successful use of a noninsulated epilating needle for intralesional desiccation in over 30 SH lesions, achieving excellent results without scarring or recurrence during a 7‐month follow‐up. Our study further validates the principle of intralesional electrothermolysis but advances it through technological refinement. While their technique utilized noninsulated needles for direct desiccation of sebaceous lobules, we employed a proximally insulated single‐needle radiofrequency probe that precisely confines energy to the dermal sebaceous units, minimizing epidermal collateral damage. This innovation aligns with the selective destruction mechanism observed in Bader's work but enhances safety via controlled depth penetration (achieved by the T‐shaped limiter) and standardized energy delivery. Briefly, single‐needle radiofrequency represents a form of intralesional electrocoagulation, wherein the proximal part of the single needle is isolated, allowing the radiofrequency energy to bypass the epidermis, thereby destroying only the targeted dermal microcomponents [15]. This treatment approach has been successfully applied to address common facial conditions, including acne [16, 17], sebaceous gland aberrance [15], and syringoma [18].

In this study, single‐needle radiofrequency was applied in the treatment of facial SH, and 97.8% of 45 patients achieved an optimal treatment response. Most patients underwent 2–3 treatment cycles, a frequency correlated with the duration and number of skin lesions in the patient population. Males required more treatment sessions and higher pulse widths. Simultaneously, the treatment demonstrated lower pain levels, quicker recovery, no risk of scarring or pigmentation changes, and a low recurrence rate of 2.2% during the 6‐month follow‐up after treatment. In a previous study conducted by Atas and Gönül [19], an excellent response (76%–100%) was observed in 65.9% of SH patients treated with cryosurgery, with no recurrence observed at the 4‐month follow‐up. Aghassi et al. [20] conducted a study on 10 immunocompetent patients using PDL for the treatment of SH. After an 8‐week follow‐up, they observed complete resolution in 28% of the lesions, a 66% decrease in diameter, and 93% flattening of the lesions. Nevertheless, 28% of the lesions reappeared after initial involution, and 7% recurred completely during the 8‐week follow‐up period. Richey and Hopson [21] detailed a cohort of 10 SH patients who underwent photodynamic therapy (PDT). In this group, 70% of lesions were successfully cleared, but 10% to 20% of treated lesions recurred within 3 to 4 months following the final treatment. High‐frequency focused ultrasound (HIFU) has recently been explored as a noninvasive option, with Woźniak et al. [22] demonstrating 87.9% complete lesion clearance in 33 SH lesions using 20 MHz HIFU, but 12.1% showed only partial response, with no long‐term recurrence data provided. The present study demonstrated superior efficacy and a lower recurrence rate in a longer follow‐up period of single‐needle radiofrequency treatment for SH, as compared to previous studies.

Dermoscopy of SH reveals the presence of white‐yellowish globules concentrated at the center of the lesion, surrounded by vessels [7]. This non‐invasive technique not only significantly enhances the accuracy of diagnosing various skin lesions but also serves as an effective means for monitoring therapeutic responses. In our study, dermoscopy demonstrated a notable reduction in both white‐yellowish globules and vessels following treatment. A comparison of dermoscopic images of the skin lesions before and after treatment enables an objective assessment of the treatment effect and the patients' recovery.

It is worth noting that our study has several limitations. First, the sample size was relatively small, potentially limiting the generalizability of our findings. Second, no traditional treatment protocols were established to compare treatment outcomes. Further expansion of the sample size and refinement of the protocol design are necessary to validate our findings.

## 5 Conclusion

In conclusion, the present study suggests that single‐needle radiofrequency is an effective and safe treatment modality for SH, with minimal adverse reactions and a low recurrence rate during the 6‐month follow‐up. Male patients exhibited with significant longer courses of disease and significantly more numbers of lesions of SH. In addition, the number of treatment sessions exhibited a positive correlation with the duration of the patient's disease and the number of lesions. The incorporation of dermoscopy in conjunction with the treatment allows for an objective evaluation of therapeutic outcomes and recovery, rendering it a valuable approach for clinical promotion.

## Author Contributions

**Xiaoyan Qiu:** conceptualization, data curation, resources; writing – original draft. **Yangyang Qiu:** conceptualization, data curation, resources, writing – original draft. **Meiqing Chen:** data curation. **Ling Huang:** data curation. **Xinyu Lin:** data curation. **Yi Wei:** data curation. **Ji Yang:** data curation. **Yuting Chen:** formal analysis, methodology, project administration, resources, validation, writing – review and editing. **Lujuan Gao:** data curation, writing – review and editing. All authors have read and approved the final manuscript. All authors have read and approved the final version of the manuscript. Lujuan Gao had full access to all of the data in this study and takes complete responsibility for the integrity of the data and the accuracy of the data analysis.

## Disclosure

Transparency statement: Lujuan Gao affirms that this manuscript is an honest, accurate, and transparent account of the study being reported; that no important aspects of the study have been omitted; and that any discrepancies from the study as planned (and, if relevant, registered) have been explained.

## Ethics Statement

This retrospective study was conducted at the department of dermatology, Zhongshan Hospital (Xiamen), Fudan University, Xiamen, China, after obtaining approval from the hospital's ethics committee. The approval number is B2024‐002.

## Consent

As routine clinical practice, informed consent was obtained from all patients before invasive treatment. Lujuan Gao hereby grants Journal of Cosmetic Dermatology permission to use the attached photo in the publication of the article titled ‘Evaluation of the efficacy and safety of single‐needle radiofrequency in patients with facial sebaceous hyperplasia: A Retrospective Study’.

## Conflicts of Interest

The authors declare no conflicts of interest.

## Data Availability

The authors confirm that the data supporting the findings of this study are available within the article.
